# Fully Automated Robust System to Detect Retinal Edema, Central Serous Chorioretinopathy, and Age Related Macular Degeneration from Optical Coherence Tomography Images

**DOI:** 10.1155/2017/7148245

**Published:** 2017-03-23

**Authors:** Samina Khalid, M. Usman Akram, Taimur Hassan, Ammara Nasim, Amina Jameel

**Affiliations:** ^1^Department of Computer Science & Information Technology, Mirpur University of Science and Technology, Mirpur, Pakistan; ^2^Department of Software Engineering, Bahria University, Islamabad, Pakistan; ^3^Department of Computer Engineering, National University of Sciences and Technology, Islamabad, Pakistan; ^4^Department of Electrical Engineering, Bahria University, Islamabad, Pakistan; ^5^Department of Computer Engineering, Bahria University, Islamabad, Pakistan

## Abstract

Maculopathy is the excessive damage to macula that leads to blindness. It mostly occurs due to retinal edema (RE), central serous chorioretinopathy (CSCR), or age related macular degeneration (ARMD). Optical coherence tomography (OCT) imaging is the latest eye testing technique that can detect these syndromes in early stages. Many researchers have used OCT images to detect retinal abnormalities. However, to the best of our knowledge, no research that presents a fully automated system to detect all of these macular syndromes is reported. This paper presents the world's first ever decision support system to automatically detect RE, CSCR, and ARMD retinal pathologies and healthy retina from OCT images. The automated disease diagnosis in our proposed system is based on multilayered support vector machines (SVM) classifier trained on 40 labeled OCT scans (10 healthy, 10 RE, 10 CSCR, and 10 ARMD). After training, SVM forms an accurate decision about the type of retinal pathology using 9 extracted features. We have tested our proposed system on 2819 OCT scans (1437 healthy, 640 RE, and 742 CSCR) of 502 patients from two different datasets and our proposed system correctly diagnosed 2817/2819 subjects with the accuracy, sensitivity, and specificity ratings of 99.92%, 100%, and 99.86%, respectively.

## 1. Introduction

The retina is the innermost layer of an eye that is composed of macular and ocular regions. The macular region is the one where inverted vision is formed. Retinal abnormalities within the macular region (maculopathy) tend to damage the macula resulting in distorted vision. These macular diseases are the collective group of abnormalities which affect the central vision of a person if they are left untreated. In extreme cases, these diseases can lead to severe visual impairments or even blindness. The major cause of these retinal abnormalities is diabetes due to which blood vessels within choroidal pathology become thinned and start leaking fluid within the intraretinal region [1]. Due to poor health infrastructure in third-world countries like Pakistan, the rate of blindness is growing yearly [2]. Also, macular disorders are the second most leading cause of blindness worldwide following cataract [3]. The most common types of these macular diseases are retinal or macular edema (ME), CSCR, and ARMD. These diseases can be easily cured if they are detected in early stages; however, due to the ignorance and unawareness in Pakistan especially in rural areas, more than 2 million people are suffering from blindness. In order to compensate this loss, a fully automated diagnostic system is required which can efficiently detect and diagnose retinal abnormalities. The diagnostic system can also act as an aid to ophthalmologists to mass-screen retinal diseases across different areas of Pakistan.

**Figure 1:**
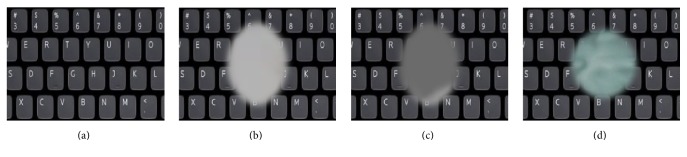
Images of (a) healthy eye vision, (b) RE affected vision, (c) CSCR affected vision, and (d) ARMD affected vision.

ME or RE mostly occurs due to leakage of fluid within retinal layers and leads to the formation of cyst spaces. CSCR or central serous retinopathy (CSR) occurs due to storage of serous fluid beneath neurosensory retina after intercepting retinal pigment epithelium (RPE) layer. CSCR is characterized into two stages. In type 1 CSCR, neurosensory retina remains intact while serous fluid gets accumulated in between RPE and neurosensory retina [4]. In type 2 CSCR, serous fluid breaches the RPE layer and gets accumulated within retinal pathology. ARMD highly correlates with aging and is primarily caused due to the formation of cellular debris, also known as drusen, within retinal and choroidal pathology. ARMD is mainly characterized into two types. The first form of ARMD, also known as dry ARMD, is related to the formation of drusen within the retinal and choroidal boundary that leads to the atrophy and degeneration of RPE. The second form of ARMD, also known as wet ARMD, is more severe and it is caused due to the formation of irregular blood vessels within choroid which intercepts retinal boundary causing severe visual impairments. This condition is also known as choroidal neovascularization (CNV). The symptoms of these diseases usually do not appear in early stages on fundus images. However, OCT imaging can easily detect the presence of these retinal abnormalities in early stages. These diseases can cause blurred and distorted central vision [5, 6] as shown in Figure 1.

**Figure 2:**
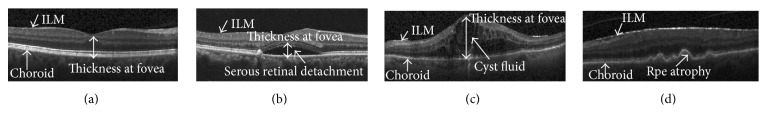
Clinical macular analysis using OCT images: (a) healthy OCT scan, (b) CSCR affected OCT scan, (c) OCT scan with RE symptoms, and (d) OCT scan with ARMD symptoms (drusen).

OCT imaging is the primary eye testing technique that is being used nowadays to detect early symptoms of retinal pathology. The major benefit of OCT over other eye testing techniques is that it can give an objective and accurate visualization of early retinal syndromes [7]. OCT imaging works on the principle of Michelson interferometer where a beam splitter decomposes low coherence light source into two parts. One part is reflected by the reference mirror and the other part goes to the candidate eye. Both parts are then merged together and form an axial scan (A-scan) [8]. The combination of multiple A-scans along the horizontal axis produces a brightness scan (B-scan).  Figure 2 shows an OCT B-scan which depicts each of the retinal syndromes along with its distinctive features.

Different scholars have clinically diagnosed RE, CSCR, and ARMD using OCT scans. Helmy and Atta Allah [9] proposed OCT based classification of cystoid macular edema (CME). Their dataset included 104 eyes of 86 test subjects and they concluded that quantitatively OCT provides better characterization of CME. Mitarai et al. [10] found alterations at leakage points in OCT scan of CSCR patients. The dataset included 23 male and 3 female subjects. It was hence concluded that, in case of CSCR, OCT detects the retinal morphological variations effortlessly. Ahlers et al. [11] discussed the variations in the retinal layers within OCT scans of CSCR affected patient. Their dataset consisted of 18 CSCR patients and it was concluded that the OCT is the noninvasive technique that can provide an objective evaluation of retinal pathology. Zhang et al. [12] presented the usage of OCT in the early detection of diabetic macular edema (DME). Teke et al. [13] presented a comparison of fluorescein angiography (FA) and OCT for the evaluation of abnormalities in 100 CSCR patients and they proved that both techniques can assist clinicians in CSCR diagnosis. Shrestha et al. [14] highlighted the importance of OCT imaging in aligning macula after ME surgery. 60 patients were involved as test subjects in their study. Ferrara et al. [15] characterized the distinguishing features of choroid and RPE that appears in OCT scans of CSCR patients. Their dataset consisted of 15 eyes of 13 patients. Mokwa et al. [16] diagnosed ARMD and CNV using FA, fundus photography (FP), and OCT imaging technique. According to their results, it was determined that fundus photography best specifies the drusen and RPE variations in case of ARMD. However, in case of CNV, OCT tends to detect minute changes better than other techniques. Wani et al. [17] presented a detailed analysis for the diagnosis of CSCR on 48 eyes and they proved that OCT is an effective technique that can replace FA for the diagnosis of CSCR. Hannouche and Ávila [18] carried out a detailed analysis between OCT, biomicroscopy, fundography, and FA to diagnose diabetic foveal edema where they concluded that OCT is capable of giving objective evaluation regarding severity of maculopathy as compared to other techniques.

Different scholars have also presented automated systems to detect retinal syndromes. Sugmk et al. [19] presented an autonomous system to diagnose ARMD and DME pathology by extracted RPE and cyst profile. To extract RPE, they removed retinal nerve fiber layer (RNFL) from the candidate B-scan where their proposed system achieved an overall accuracy of 100% for ARMD cases and 86.6% for DME cases. Srinivasan et al. [20] presented a fully automated system for the diagnosis of ARMD, DME, and healthy subjects from OCT B-scans. They utilized histogram oriented gradients (HOG) feature descriptor to extract meaningful features and then they used SVM for the automated diagnosis of retinal pathology. The accuracy of their proposed system was 100% for both ARMD and DME subjects and 86.67% for healthy subjects. Zhang et al. [23] utilized adaptive boosting (AdaBoost) based classification system for the automated diagnosis of cystoid macular edema (CME) through segmentation of intraretinal layers. The accuracy of their proposed system was 98.6%. Wilkins et al. [22] used manual annotations of RPE and ILM to identify the fluid within retinal layers. They evaluated their proposed system on 16 test subjects with overall 91% sensitivity and 96% specificity. To the best of our knowledge, there is no technical paper available that gives automated detection of ME, CSCR, and ARMD from OCT scans.

Previously, we have proposed a fully automated robust system in [24] to diagnose ME, CSR, and normal cases from 2D OCT scans and here we propose an extension of our system to incorporate automated detection and diagnoses of macular disorders. As there are different macular diseases, that is, RE, CSCR, and ARMD, which show a little bit similar variations in OCT scans near the fovea, while designing an automated system for detection of any macular disease, it is important to differentiate between these macular disorders. This is the main contribution we have made in this article that presents a fully automated decision support system to detect RE, CSCR, and ARMD from OCT B-scans. The automated classification in our proposed system is based on extracting 9 distinct features from the coherent tensor of candidate OCT scan, and then these features are fed to multilayered SVM classifiers to automatically diagnose CSCR, ME, or ARMD retinal subjects.

The rest of the paper is arranged in a way that Section 2 demonstrates the proposed methodology and Section 3 depicts the results while Section 4 outlines the conclusion of the paper.

## 2. The Proposed Methodology

**Figure 3:**
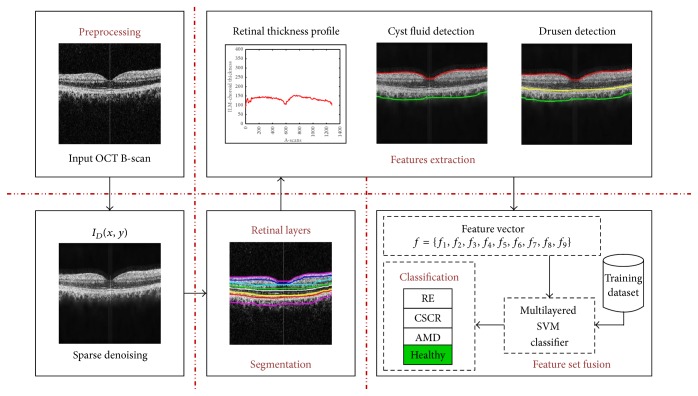
Detailed step-by-step flow diagram of the proposed system.

An autonomous decision support system is proposed here for the automated self-diagnosis of RE, CSCR, and ARMD pathology from OCT images. At first, the input OCT scan *I*(*s*, *t*) is loaded into our proposed system which is denoised using adaptive Wiener filter. The objective of denoising the candidate scan is to increase the sparsity within intraretinal pathology. After denoising the candidate scan, we extracted intraretinal layers to discriminate between normal and abnormal retinal pathology. These retinal and choroidal layers are segmented by computing a highly coherent tensor representation of macular pathology [25]. Extracted inner limiting membrane (ILM) and choroidal layer are used to compute cyst pathology within the candidate scan. Drusen within the retinal and choroidal boundary are detected by extracting RPE and measuring atrophy and retinal degeneration. After that, a 9D feature vector is obtained based on retinal thickness and cyst profile, RPE atrophic profile, and drusen. The feature vector is then passed to the trained multilayered SVM classifier to diagnose the retinal syndrome.  Figure 3 shows the block diagram of our proposed system.

*OCT Dataset.* There are two datasets that have been in this research. The first one is our local dataset that contains 90 OCT B-scans (30 healthy, 30 RE, and 30 ARMD) acquired from 73 patients (19 females and 54 males). This dataset has been acquired from AFIO, Rawalpindi, and the OCT images within the dataset are captured using TOPCON 3D OCT-2000 machine. The detailed description of the dataset is shown in Table 1. Apart from this, we have also used the publicly available Duke dataset that contains 2729 OCT images (1407 healthy, 610 RE, and 712 ARMD) of 429 patients. Duke dataset has been acquired from Spectralis spectral domain optical coherence tomography (SD-OCT) imaging camera. The manufacturer of Spectralis SD-OCT machine is Heidelberg Engineering Inc. Apart from this, Duke dataset (used in [20, 26]) has been annotated by multiple expert ophthalmologists and it is publicly available online at the following links:

 
http://people.duke.edu/~sf59/Srinivasan_BOE_2014_Dataset.htm
 
http://people.duke.edu/~sf59/RPEDC_Ophth_2013_dataset.htm

### 2.1. Preprocessing

An input OCT scan *I*(*s*, *t*) is initially loaded into our proposed system and if it is a color image then only the highest intensity contributing channel *I*_Gray_(*s*, *t*) is kept for further processing. Afterwards, *I*_Gray_(*s*, *t*) is normalized to the common spatial resolution of 480 × 1280. Then, it is denoised using 2D adaptive low pass Wiener filter. The reason for denoising the candidate image is to increase the sparsity of intraretinal pathology within *I*_Gray_(*s*, *t*). Wiener filter adaptively suppresses noisy outliers by measuring an average intensity of the surrounding pixels within the filtering kernel as expressed in

(1) ℶ=1wswt∑sa∈ws ∑tb∈wtIsa,tbℵ2=1ωsωt∑sa∈ws ∑tb∈wsI2sa,tb−ℶ2IDsa,tb=ℶ+ℵ2−φ2ℵ2Isa,tb−ℶ,

where *I*_*D*_(*s*_*a*_, *t*_*b*_) represents the sparsely strained pixel, *w*_*s*_ represents the row of a smoothing window, *w*_*t*_ represents the column of the smoothing window, *ℶ* represents the localized mean within the kernel, *ℵ*^2^ represents the localized variance within the kernel, and *φ*^2^ is the mean of all *ℵ*^2^ kernels [27].

### 2.2. Retinal Layers Segmentation

In order to segment retinal pathology from the candidate scan, a second-order structure tensor grid is computed in our proposed system that takes a candidate denoised scan *I*_*D*_(*s*, *t*) and generates its partial derivatives at the orientation of 0 and *π*/2 radians. Since the gradients are computed along two predominant orientations, these gradients are fused together to generate four possible tensors as expressed mathematically in

(2) Is,tΓSSs,tΓSTs,tΓTSs,tΓTTs,tΓSSs,t∑si∈ζx ∑tj∈ζjξsi,tjΔSSs−si,t−tjΓSTs,tΓTSs,t=∑si∈ζx ∑tj∈ζjξsi,tjΔSTs−si,t−tjΓTTs,t∑si∈ζx ∑tj∈ζjξsi,tjΔTTs−si,t−tj,

where *ℑ*(*s*, *t*) represents a second-order structure tensor matrix containing all possible tensors among two predominant orientations and Γ_*SS*_(*s*, *t*), Γ_*ST*_(*s*, *t*), Γ_*TS*_(*s*, *t*), and Γ_*TT*_(*s*, *t*) represent the convolution sum of gradient products at the respective orientation [28]. The gradient products (Δ_*SS*_, Δ_*ST*_, Δ_*TS*_, and Δ_*TT*_) are mathematically expressed in

(3) ΔSS=IDSSs,t2ΔST=IDSs,t·IDTs,tΔTT=IDTTs,t2.

**Figure 4:**
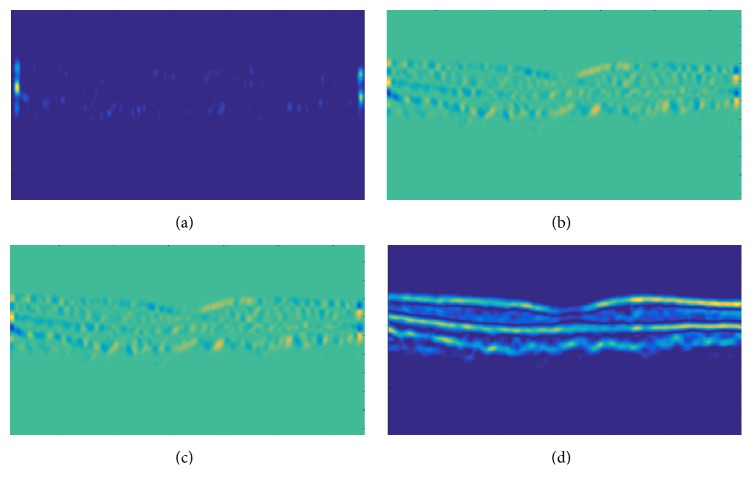
Second-order structure tensor grid: (a) tensor computed through the dot product of horizontal gradient, (b) tensor computed through the dot product of horizontal and vertical gradients, (c) tensor computed through the dot product of vertical and horizontal gradients, and (d) tensor computed through the dot product of vertical gradient.

In order to smoothen each tensor within the tensor grid, a localized Gaussian window *ξ*(*s*, *t*) is computed which is convolved with the gradient products. Out of these tensors, a highly coherent tensor is obtained which has the maximum coherency (*ℌ*). *ℌ* is computed using

(4) $$\begin{array}{c} H={\left(\;\frac{{ℷ}_{1}-{ℷ}_{2}}{{ℷ}_{1}+{ℷ}_{2}}\;\right)}^{2}, \end{array}$$

where *ℷ*_1_ and *ℷ*_2_ represent the eigenvalues of partial derivatives computed along 0 and *π*/2 radians. The computed tensors are shown in Figure 4.

**Figure 5:**
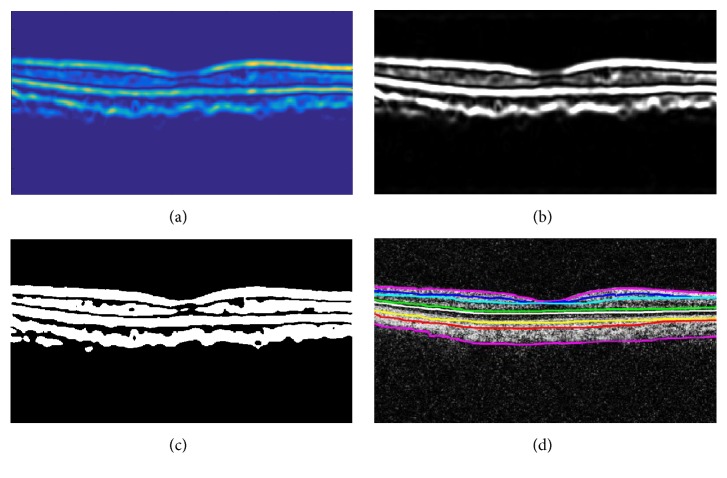
Segmented retinal and choroidal layers: (a) highly coherent 2D structure tensor *I*_*C*_(*s*, *t*), (b) binary map *I*_*B*′_(*s*, *t*) of highly coherent tensor, (c) canny edge detection of retinal and choroid layer, and (d) segmented retinal layers.

After extracting the highly coherent tensor *I*_*C*_(*s*, *t*), the binary map *I*_*B*′_(*s*, *t*) of *I*_*C*_(*s*, *t*) is computed using Otsu algorithm [29]. Afterwards, retinal layers are extracted from the digitalized map *I*_*B*′_(*x*, *y*) by computing retinal edges using canny edge detection [30], as shown in Figure 5.

**Figure 6:**
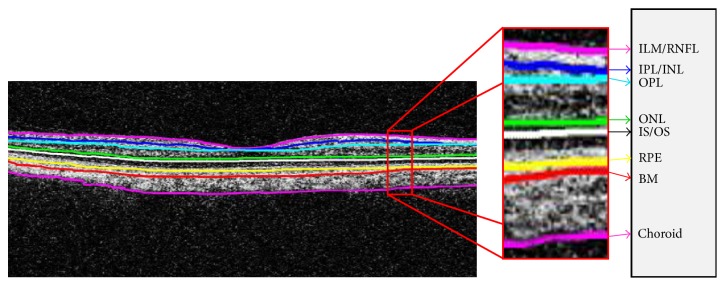
Segmented retinal layers in one B-scan: ILM, retinal nerve fiber layer (RNFL), inner plexiform layer (IPL), inner nuclear layer (INL), outer nuclear layer (ONL), outer plexiform layer (OPL), outer segment (OS), inner segment (IS), RPE, Bruch's membrane (BM), and choroid.

Retinal layers segmented out from the candidate scan are shown in Figure 6. It can be observed from Figure 6 that, in the case of healthy subjects, all retinal layers are closely intact without any deformity.  Table 2 shows the axial scans (A-scans) mean separation between retinal layers of all 4 types of retinal disorders. It can be observed from the tabulated data that in case of healthy subjects the mean value for all retinal layers is less as compared to CSCR and RE cases. For ARMD cases, all retinal layers are quite close to each other except for RPE which is because of the deformity in RPE layer due to ARMD pathology.

### 2.3. Features Extraction

After extracting retinal layer pathology from candidate scan, a 9D feature vector is extracted for automated disease self-diagnosis. A feature vector is a collection of 9 distinct features obtained from candidate OCT B-scan. The first 5 features are gathered by extracting retinal thickness and cyst profile from the candidate scan. The remaining 4 features are computed by extracting and analyzing RPE atrophic profile.

#### 2.3.1. Thickness Profile Extraction

**Figure 7:**
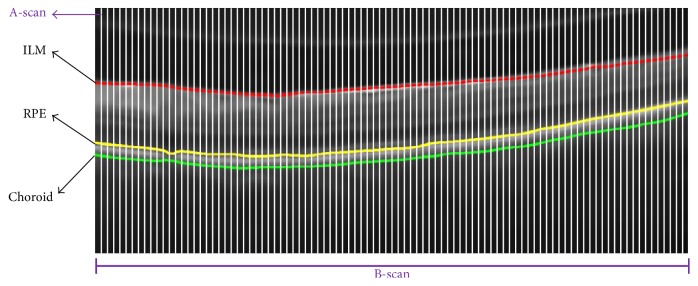
OCT scans structure. A-scan after the 10th interval is highlighted by white color. ILM is shown in red color, RPE is shown in yellow color, and choroid is highlighted in green color. A-scans were fused together to form a single B-scan.

**Figure 8:**
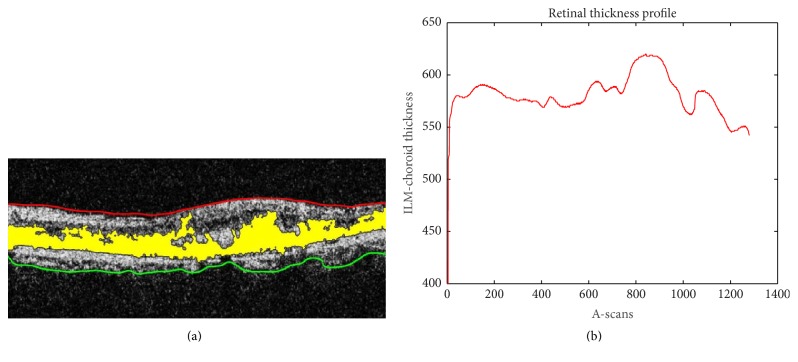
(a) Segmented ILM, choroid, and intraretinal cyst pathology mapped onto the candidate scan; (b) retinal thickness profile.

After extracting intraretinal pathology, ILM and choroidal layers are used to generate a B-scan retinal thickness profile *R*_*T*_(*t*) as illustrated by (5) and (6). Hence,

(5) $$\begin{array}{c} R_{T}\left(\;t\;\right)=\left[\;R_{T}\left(\;t_{1}\;\right),R_{T}\left(\;t_{2}\;\right),\ldots ,R_{T}\left(\;t_{i}\;\right)\;\right], \end{array}$$

where

(6) $$\begin{array}{c} R_{T}\left(\;t_{i}\;\right)=\left(\;\left|\;I_{{ILM}_{i}}\left(\;s,t\;\right)-I_{{Choroid}_{i}}\left(\;s,t\;\right)\;\right|\;\right), \end{array}$$

where “*i*” represents the number of A-scans present within a B-scan also shown in Figure 7.  Figure 8 depicts the retinal thickness profile of the candidate scan suffering from RE.

#### 2.3.2. Cyst Fluid Detection

**Figure 9:**
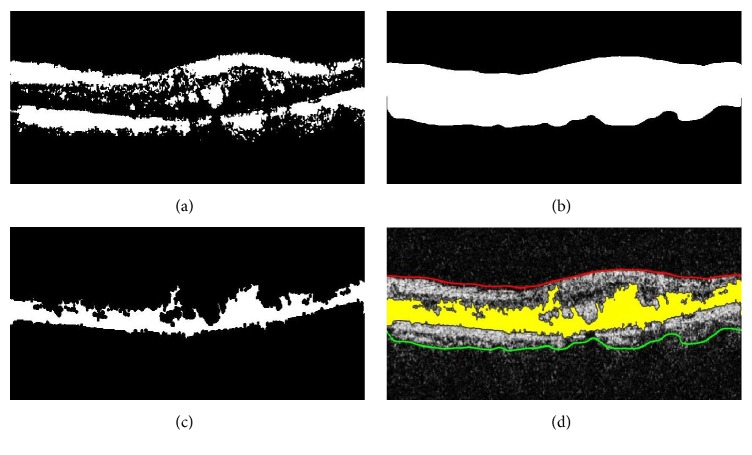
Cyst fluid detection: (a) *I*_*B*′_(*s*, *t*); (b) *R*_*M*_(*s*, *t*) generated through ILM and choroid; (c) Cyst(*s*, *t*); (d) extracted cyst fluid mapped onto the candidate scan. It is shown in yellow color.

After segmenting retinal layers, the proposed system detects cyst segments within retinal pathology. A retinal mask *R*_*M*_(*s*, *t*) is created in between ILM and choroid layer and it is logically fused with *I*_*B*′_(*s*, *t*) to extract the cyst pathology.  Figure 9 shows the exact cyst segments that are obtained using

(7) $$\begin{array}{c} Cyst\left(\;s,t\;\right)=R_{M}\left(\;s,t\;\right)⊕I_{B^{\prime }}\left(\;s,t\;\right). \end{array}$$

#### 2.3.3. Drusen Detection

**Figure 10:**
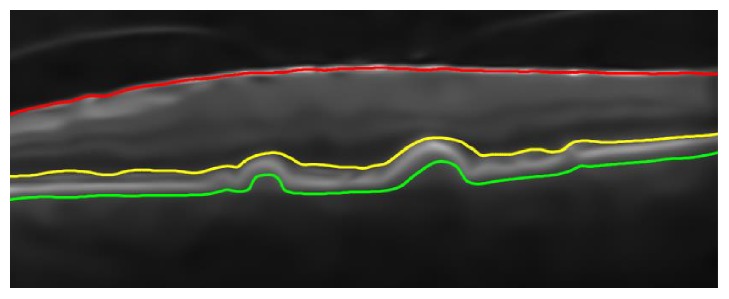
Drusen detection through atrophic analysis of RPE. Segmented ILM is shown in red color. RPE is highlighted in yellow color. Choroid is shown in green color.

In conjunction with extracting cyst pathology, our proposed system also analyzes atrophy and degeneration within the extracted RPE layer for possible detection of drusen within the retinal and choroidal boundary. If there is a deformity within the RPE layer, then this is due to the presence of drusen and neovascularization which leads to ARMD. Therefore, in our proposed system, we have extracted 4 distinct features based on RPE pathology which automatically detect the presence of ARMD syndrome as shown in Figure 10.

Apart from this, both RE and CSCR pathology have fluid accumulation within the retinal layer, so in order to discriminate between both diseases, our proposed system computes cyst energy obtained after decomposing the cyst profile into a low resolution band through multilevel wavelet decomposition technique. The value of this cyst energy is much less in case of CSCR subjects as compared to RE subjects as shown in Table 3.

### 2.4. Feature Set Fusion

After computing the individual characteristic profile for each disease, our proposed system extracts 9 distinct features from these profiles and fuses a 9D feature vector which is passed to the multilayered SVM classifier. The first 4 features are extracted from retinal B-scan thickness and cyst profile to distinguish between healthy and diseased scans. The 5th feature is used to discriminate between RE and CSCR syndromes and the last 4 features are extracted after analyzing RPE profile to distinguish ARMD cases. The detailed description of each feature is as follows.

*Max Thickness *(*f*_1_). It is actually the maxima in the retinal thickness vector *R*_*T*_(*t*), which is due to maximum difference between the ILM and choroid as expressed by

(8) f1=max⁡RTtf1=max⁡IILMs,t−IChoroids,t.

*Min Thickness *(*f*_2_). It is actually the minima in the retinal thickness vector *R*_*T*_(*t*) which is due to minimum difference between ILM and choroid layer as expressed by

(9) f2=min⁡RTtf2=min⁡IILMs,t−IChoroids,t.

*Thickness Variation *(*f*_3_). It depicts the retinal thickness variation within macular pathology and it is computed using

(10) $$\begin{array}{c} f_{3}=f_{2}-f_{1}. \end{array}$$

*Maximum Cyst Area *(*f*_4_). It depicts the maximum area occupied by the cyst or serous fluid within intraretinal pathology and it is computed by taking area of Cyst(*s*, *t*) as expressed by

(11) $$\begin{array}{c} f_{4}=Area\left(\;Cyst\left(\;s,t\;\right)\;\right). \end{array}$$

*Cyst Energy *(*f*_5_). It is the total energy of a cyst segment calculated by

(12) $$\begin{array}{c} f_{5}={\sum \left|Low\;\;band\left(DWT\left(Cyst\left(s,t\right)\right)\right)\right|}^{2}. \end{array}$$

*RPE Maxima *(*f*_6_). It is the peak value in RPE profile as expressed by

(13) $$\begin{array}{c} f_{6}=\max\left[\;I_{RPE}\left(\;t\;\right)\;\right]. \end{array}$$

*RPE Minima *(*f*_7_). It is the shallowest value in RPE profile as expressed by

(14) $$\begin{array}{c} f_{7}=\min\left[\;I_{RPE}\left(\;t\;\right)\;\right]. \end{array}$$

*RPE Variation *(*f*_8_). It is the absolute difference between *f*_7_ and *f*_6_ as expressed by

(15) $$\begin{array}{c} f_{8}=\left|\;f_{7}-f_{6}\;\right|. \end{array}$$

*RPE Energy *(*f*_9_). It depicts the RPE energy as expressed by

(16) $$\begin{array}{c} f_{9}=\overset{N-1}{\underset{i=0}{\sum }}{\left|\;I_{RPE}\left(\;i\;\right)\;\right|}^{2}. \end{array}$$

Table 3 depicts 9 distinct features extracted from 5 randomly selected samples of each class. From Table 3, we can see that the first 5 features are quite distinctive for healthy, RE, and CSCR cases while the last 4 features are quite distinctive for ARMD cases. For ARMD cases, the first 3 features are quite close to healthy cases; however, discrimination between healthy and ARMD cases can be best seen in *f*_4_, *f*_6_, *f*_7_, *f*_8_, and *f*_9_.

### 2.5. Classification

#### 2.5.1. Classifier Training

**Figure 11:**
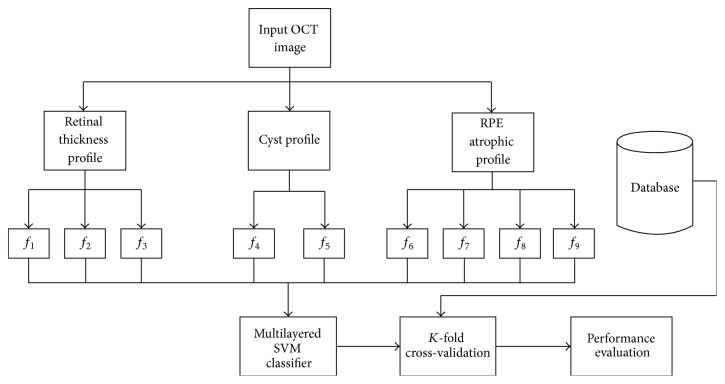
Training phase of SVM.

The classification system in our proposed system uses multilayered SVM classifier to distinguish between retinal abnormalities. After processing the input candidate OCT scan, 9 distinct features are extracted which are fused together to form a 9D feature vector *f* = {*f*_1_, *f*_2_, *f*_3_, *f*_4_, *f*_5_, *f*_6_, *f*_7_, *f*_8_, *f*_9_}. The feature vector *f* is then passed to the multilayered supervised SVM classifier for automated disease diagnosis. The classification system in our proposed system is trained on our custom prepared training dataset that includes 40 labeled images (10 healthy, 10 RE, 10 CSCR, and 10 ARMD). The dataset has been annotated by multiple expert ophthalmologists. The first 5 features in the feature set are extracted from retinal thickness and cyst profile. The remaining 4 features are acquired by analyzing atrophy within the RPE profile. SVM is being incorporated in our proposed classification system because it is one of the fastest and accurate classifiers [31]. In our proposed system, SVM has a nonlinear decision boundary because of Gaussian radial basis function (RBF) and multilayer perceptron (MLP) kernel.  Figure 11 demonstrates the training phase of our proposed classification system.

The performance of our classification system is measured through *K*-fold cross-validation. We cross-validated our classification system for different values of *k* and computed the accuracy. The best accuracy was achieved for *k* = 10 as shown in Table 4.

#### 2.5.2. Classification of Retinal Pathologies

**Figure 12:**
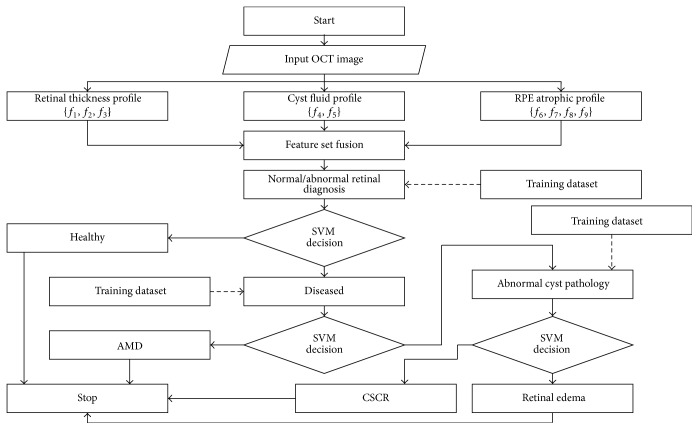
Flowchart of classification algorithm.

After training the proposed classification system, it was used to classify unlabeled input OCT scan for possible diagnosis of retinal abnormalities. The classification system in our proposed system is based on multilayered SVM classifier in which the test sample is first classified as healthy or abnormal through *f*_1_, *f*_2_, *f*_3_, and *f*_4_ features. If it is classified as abnormal, then it is further classified as RE, CSCR, or ARMD positive candidate based upon the remaining features within the feature vector. SVM in our proposed system has been implemented in 3 layers to classify all 4 types of macular syndromes. The flowchart of our proposed decision support system is shown in Figure 12.

## 3. Results

Our proposed system was tested on our local dataset which we acquired from AFIO. The dataset contained 90 OCT scans of 73 patients in which 30 images are of RE patients, 30 images are of CSCR patients, and 30 images are of healthy subjects. Apart from this, we have tested and validated our proposed system on the publicly available Duke dataset containing 2729 OCT images of 429 patients in which 712 OCT images are of ARMD patients, 610 OCT images are of RE patients, and 1407 OCT images are of healthy subjects. Our proposed system correctly identified all the retinal pathologies on Duke dataset, while on our local dataset our proposed system correctly identified 88/90 cases. The results from our automated decision support system have been cross-verified by expert ophthalmologists as well. The detailed statistical analysis of our proposed system is shown in Table 5.

**Figure 13:**
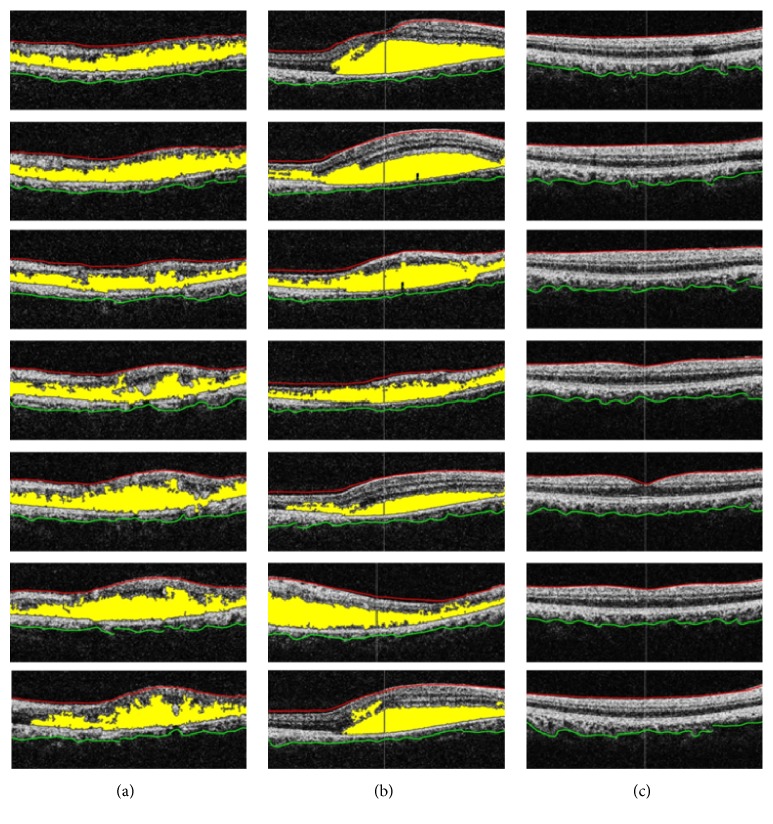
Unlabeled AFIO dataset: (a) RE classified scans; (b) CSCR classified scans; (c) healthy classified scan.

**Figure 14:**
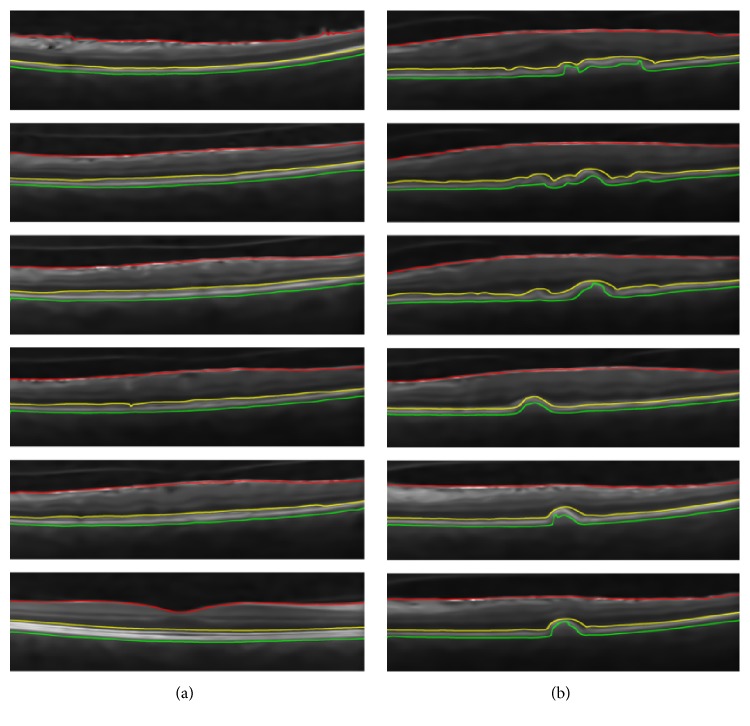
Unlabeled Duke dataset: (a) healthy classified scans; (b) ARMD classified scans.

Figure 13 shows 7 randomly selected B-scans for each pathology from AFIO dataset that has been correctly classified. In each scan, ILM is shown in red color, choroid is shown in green color, and cyst or serous pathology is shown in yellow color. Apart from this, Figure 14 shows 6 randomly selected B-scans from Duke dataset that have been correctly classified. The color scheme remains the same as in Figure 13 except for yellow color which is used to show the extracted RPE layer in Figure 14.

In addition to this, we have also compared our multilayered classification system with other state-of-the-art solutions where our proposed system has outperformed other competitors as shown in Table 6. Also, we proposed the world's first ever automated decision support system that can automatically detect 4 different types of retinal pathologies from OCT images.

## 4. Discussion

A fully automated decision support system is proposed here which can automatically detect and self-diagnose retinal abnormalities from OCT images by extracting 9 distinct features. After that, all the extracted features are fused together to form a feature vector which is then passed to the multilayered support vector machine classification system to automatically diagnose the retinal pathology. The proposed system is quite robust in detecting small disease patterns that appear on OCT images. Apart from this, our proposed system is also rotationally invariant and can easily detect the above mentioned retinal diseases from skewed OCT B-scans. Our proposed system extracts 7 to 8 intraretinal layers from all 4 types of retinal pathologies and uses ILM, RPE, and choroidal layers for the formation of cyst or serous pathology and ARMD atrophic profile.

**Figure 15:**
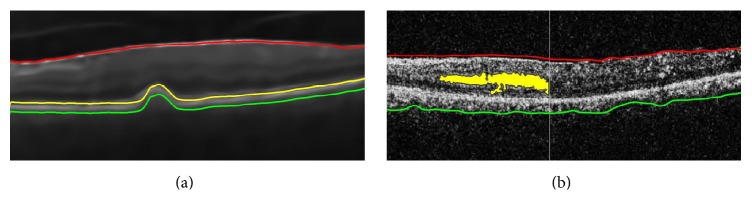
Early self-diagnosis of macular syndromes: (a) early symptoms of RPE atrophy due to the presence of drusen; (b) early formation of cyst fluid within macular pathology. These scans are correctly identified by our proposed system.

The proposed multilayered classification system is based on 9D feature vector, extracted from candidate OCT B-scan. At the first layer of our classification system, an automated decision between normal and abnormal retinal pathology is made by analyzing *f*_1_, *f*_2_, *f*_3_, and *f*_4_ as these features contain the objective evaluation of retinal thickness profile. If the candidate is classified as abnormal, then it is further classified as RE, CSCR, and ARMD. The discrimination between RE cysts and CSCR is provided by *f*_5_ features as RE cysts contain more energy as compared to CSCR. Also, the presence of drusen within the retinal and choroidal boundary is detected by atrophic analysis of RPE profile through *f*_6_, *f*_7_, *f*_8_, and *f*_9_ features. All of these features for 5 randomly selected subjects from each case are shown in Table 3. Apart from this, we have applied our proposed system on our local dataset acquired from AFIO and also publicly available Duke dataset. AFIO dataset contains samples of RE, CSCR, and healthy subjects while Duke dataset contains samples of RE, ARMD, and healthy subjects. Our proposed system correctly classifies a total of 2817/2819 retinal pathologies from both datasets. Detailed analysis of results is shown in Table 5. The misclassification of two healthy samples as diseased from AFIO dataset is because we have tuned our system in such a way to give more weightage to the correct classification of diseased samples as it is more critical to classify diseased samples accurately as compared to healthy subjects. Our proposed system is also computationally quite fast and it takes around a minute on average to give a complete disease diagnosis on a machine with 5th-generation core i5 CPU (2.2 GHz) and 4 GB DDR3 RAM. Our proposed system is quite robust and sensitive to retinal abnormalities as it can also detect small and early retinal abnormalities from OCT B-scan. Two of such cases are shown in Figure 15.

Our automated self-diagnosis decision support system can act as an aid to ophthalmologists to mass-screen the severe cases of retinal abnormalities. Also, our proposed system can give an objective disease diagnosis with complete statistical analysis of retinal pathology which ophthalmologists can use to back up their diagnosis. Based on the statistical results obtained from our proposed system, ophthalmologists can improve their standardized grading system for the severity analysis of different retinal pathologies.

## 5. Conclusion

This paper proposes fully automated self-diagnosis system to identify healthy, RE, CSCR, and ARMD cases from OCT images. The automated classification of candidate retinal pathology is based on multilayered SVM classifier that is trained to distinguish between all 4 types of macular syndromes by analyzing 9 different, unique, and distinct features. These features are extracted by first segmenting intraretinal and choroidal layers from candidate OCT B-scan and then by computing retinal thickness profile, cyst or serous pathology, and atrophic RPE profile. Our proposed system was applied on 90 OCT B-scans from AFIO dataset and 2729 OCT B-scans from Duke dataset where our system correctly identified 2729/2729 samples from Duke dataset and 88/90 samples from AFIO dataset.

Apart from this, our proposed system was quite robust in detecting small abnormalities within macular pathology from noisy and skewed OCT B-scans. In the future, this work can be extended to automatically diagnose the severity of these macular syndromes; also, our proposed system can be extended to incorporate other retinal abnormalities like tractional retinal detachment (TRD), macular hole (MH), and glaucoma.

## Figures and Tables

**Table 1:** Scanning parameters of AFIO dataset.

Scanning parameters	Type		
	Healthy	CSCR	RE
Total subjects	30	30	30
Axial resolution (um)	3~3.8	3~3.8	3~3.8
Lateral resolution (um)	11~13	7~13	11~13
Azimuthal resolution (um)	49~122	58~129	63~186
Scan resolution (pixel × pixel)	480 × 1280	480 × 1280	480 × 1280
B-scans	128	115~134	117~126
A-scans (points)	1280 points	1280 points	1280 points

**Table 2:** Retinal layers separation for all 4 types of retinal subjects.

Layers	Healthy	CSCR	RE	ARMD
	Mean ± SD (*µ*m)	Mean ± SD (*µ*m)	Mean ± SD (*µ*m)	Mean ± SD (*µ*m)
ILM-IPL/INL	36.789 ± 6.21	67.248 ± 10.29	84.215 ± 14.71	35.475 ± 5.31
IPL/INL-OPL	38.114 ± 5.47	54.764 ± 13.17	124.10 ± 16.68	34.701 ± 7.83
OPL-ONL	40.028 ± 8.13	74.599 ± 15.85	115.738 ± 15.92	36.807 ± 7.63
ONL-IS/OS	29.437 ± 3.64	248.168 ± 32.64	192.577 ± 21.96	43.675 ± 2.54
IS/OS-RPE	32.582 ± 5.82	161.573 ± 24.91	201.612 ± 24.09	143.524 ± 27.39
RPE-BM	31.937 ± 4.86	60.008 ± 15.08	108.349 ± 14.79	187.237 ± 23.16
BM-choroid	36.768 ± 5.19	64.386 ± 9.78	94.02 ± 15.51	31.524 ± 4.13

**Table 3:** Feature vectors from 5 randomly selected subjects from each category.

Type	Cases	Features								
		*F*1	*F*2	*F*3	*F*4	*F*5	*F*6	*F*7	*F*8	*F*9
		(mm)	(mm)	(mm)	(mm^2^)		(mm)	(mm)	(mm)	
Healthy	Case 1	36.51	11.38	25.13	124.27	1165.2	1.20	−0.36	1.56	24.070
	Case 2	39.69	27.02	12.67	140.28	1328.8	0.87	−0.80	1.67	29.060
	Case 3	34.13	19.58	14.55	0	0	0.80	−0.57	1.37	26.008
	Case 4	50.54	32.55	17.99	126.71	1124.1	4.00	−2.56	6.56	68.206
	Case 5	38.36	18.26	20.10	174.34	983.24	0.93	−0.36	1.29	32.036
	*Mean*	*39.84*	*21.75*	*15.09*	*113.12*	*920.26*	*1.56*	*−0.93*	*2.49*	*35.87*
	*STD*	*6.33*	*8.19*	*4.02*	*66.31*	*528.98*	*1.37*	*0.92*	*2.28*	*18.32*

CSCR	Case 1	64.29	38.1	26.19	11827	10417	1.40	−0.26	1.66	32.010
	Case 2	82.02	42.33	39.69	20981	8570.6	1.72	−0.79	2.51	31.050
	Case 3	68.79	34.66	34.13	22936	12061	0.76	−0.65	1.41	27.038
	Case 4	63.5	43.39	20.11	8724.1	12940	3.96	−3.12	7.08	78.519
	Case 5	71.7	35.98	35.72	26443	13051	0.89	−0.65	1.54	42.213
	*Mean*	*70.06*	*38.89*	*31.16*	*18183*	*11408*	*1.74*	*−1.09*	*2.84*	*42.16*
	*STD*	*7.47*	*3.84*	*7.89*	*7558.6*	*1904.3*	*1.29*	*1.14*	*2.40*	*21.07*

RE	Case 1	72.23	36.25	35.98	7023.8	56835	1.32	−0.56	1.88	42.030
	Case 2	48.42	25.66	22.76	18400	32093	1.75	−0.49	2.24	31.430
	Case 3	49.48	21.96	27.52	14619	23180	0.72	−0.75	1.47	37.148
	Case 4	62.18	33.07	29.11	27120	27419	4.93	−2.42	7.35	58.159
	Case 5	65.62	39.43	26.19	3201.17	25871	0.69	−0.15	0.84	39.535
	*Mean*	*59.58*	*31.27*	*28.31*	*14073*	*33080*	*1.88*	*−0.87*	*2.75*	*41.66*
	*STD*	*10.36*	*7.29*	*4.88*	*9449.4*	*1366.8*	*1.76*	*0.89*	*2.62*	*10.02*

ARMD	Case 1	32.83	16.25	16.58	1523.	1147.1	10.93	−1.20	12.13	118.47
	Case 2	38.22	25.36	12.86	2040.9	1452.7	13.08	−1.64	14.72	207.68
	Case 3	39.18	16.66	22.52	1534.1	1365.2	16.48	−5.60	22.08	439.89
	Case 4	47.58	30.37	17.21	1670.2	1678.3	11.37	−2.35	13.72	140.38
	Case 5	35.72	12.22	23.50	2019.5	417.26	11.93	−0.88	12.81	106.29
	*Mean*	*38.70*	*20.17*	*18.53*	*1757.7*	*1212.1*	*12.75*	*−2.33*	*15.09*	*202.54*
	*STD*	*5.53*	*7.44*	*4.42*	*255.47*	*483.39*	*2.23*	*1.90*	*4.02*	*138.34*

**Table 4:** Classifier cross-validation performance.

*K*	Max. accuracy
2	92.4%
4	94.8%
8	95.7%
**10**	**98.2%**
12	96.5%

**Table 5:** Results achieved.

Dataset	Type	Correctly classified	Accuracy	Sensitivity	Specificity
AFIO	Healthy	28/30	97.77%	100%	93.33%
	CSCR	30/30			
	RE	30/30			

Duke	Healthy	1407/1407	100%	100%	100%
	RE	610/610			
	ARMD	712/712			

Total	Healthy	1435/1437	99.92%	100%	99.86%
	RE	640/640			
	ARMD	742/742			

**Table 6:** Classifier performance comparison.

Authors	Pathologies	Dataset	Accuracy	Sensitivity	Specificity
*Proposed*	*4*	*2819*	*99.92%*	*100%*	*99.86%*
[19]	2	16	—	91%	96%
[20]	3	16	87.5%	—	—
[21]	2	550	84%	93	80
[22]	3	45	95.5	100	93.75
